# QuantIF: An ImageJ Macro to Automatically Determine the Percentage of Infected Cells after Immunofluorescence

**DOI:** 10.3390/v11020165

**Published:** 2019-02-19

**Authors:** Lynda Handala, Tony Fiore, Yves Rouillé, Francois Helle

**Affiliations:** 1 EA4294, Agents Infectieux, Résistance et Chimiothérapie, Centre Universitaire de Recherche en Santé, Centre Hospitalier Universitaire et Université de Picardie Jules Verne, 80054 Amiens, France; l.handala@gmail.com (L.H.); toni.fiore37@gmail.com (T.F.); 2 University of Lille, CNRS, INSERM, CHU Lille, Pasteur Institute of Lille, U1019-UMR8204-CIIL-Center for Infection and Immunity of Lille, 59019 Lille, France

**Keywords:** virus, infection, fluorescent reporter protein, image quantification, Hepatitis C virus, Yellow Fever Virus, polyomavirus, Coxsackievirus B4

## Abstract

Counting labeled cells, after immunofluorescence or expression of a genetically fluorescent reporter protein, is frequently used to quantify viral infection. However, this can be very tedious without a high content screening apparatus. For this reason, we have developed QuantIF, an ImageJ macro that automatically determines the total number of cells and the number of labeled cells from two images of the same field, using DAPI- and specific-stainings, respectively. QuantIF can automatically analyze hundreds of images, taking approximately one second for each field. It is freely available as supplementary data online at MDPI.com and has been developed using ImageJ, a free image processing program that can run on any computer with a Java virtual machine, which is distributed for Windows, Mac, and Linux. It is routinely used in our labs to quantify viral infections in vitro, but can easily be used for other applications that require quantification of labeled cells.

## 1. Introduction

When evaluating viral infections in vitro, fluorescence microscopy is commonly used to monitor the expression of a viral protein following immunostaining. However, this method requires a high content screening apparatus to count large numbers of fluorescent cells. Manual evaluation is feasible when analyzing few images, but it can result in subjective evaluation by the researcher. Furthermore, it is very time-consuming when working with hundreds of images, containing thousands of cells per image.

ImageJ is a free image-processing program that was developed 20 years ago by Wayne S. Rasband at the National Institute of Health, and has become a valuable tool for researchers [1,2]. It is a Java-based software that can run on any computer using a Java virtual machine. It is thus available for Windows, Mac, and Linux. ImageJ can convert images into numerical values that can be exported and further processed with other software for statistical analysis. Furthermore, a major strength of ImageJ is the possibility to record macros that enable the automatization of image analysis.

In this technical note, we present QuantIF, an ImageJ macro for determining the percentage of fluorescent cells following immunofluorescence staining. QuantIF can be used when the specific staining in the cytoplasm and/or nucleus of a cell is diffuse. The macro automatically determines the total number of cells and fluorescently labeled cells for a series of images corresponding to different conditions. For each condition, two pictures of the same field must be taken, the first one corresponding to the specific staining and the second one corresponding to the DAPI staining. In this way, the series of images to be analyzed are placed in the same folder, with images corresponding to the specific staining in odd rank and images of DAPI staining in even rank. When the macro is run, it automatically processes all images in the folder, taking around one second to analyze both images of each field. Ultimately, all results are saved as a “.xls” file that can be processed for statistical analysis.

## 2. Macro Description

**Figure 1:**
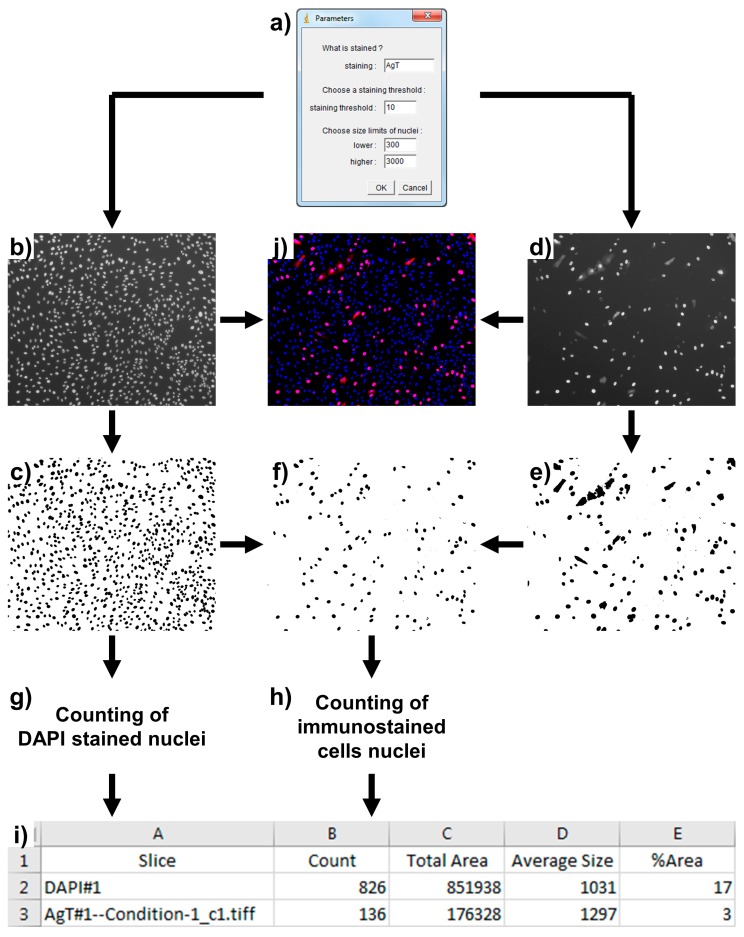
Description of the QuantIF macro. After entering the parameters into the dialog box (**a**), two images of each field are analyzed. The DAPI staining image (**b**) is converted to a DAPI staining mask (**c**), and the specific staining image (**d**) is converted to a specific staining mask (**e**), by implementing the Huang’s fuzzy thresholding method. A third mask corresponding to the nuclei of the immunostained cells is created using the “Image Calculator” command and the “AND” operator (**f**). Finally, DAPI stained nuclei and immunostained cell nuclei are counted using the “Analyze Particles” tool (**g**,**h**). After processing, the numbers of DAPI-stained nuclei and immunostained cell nuclei for each condition are saved as a “.xls” file in the folder that has been analyzed (**i**). A merge of the DAPI and specific staining images is shown for informational purposes (**j**).

QuantIF was developed using ImageJ version 1.52e and Java version 8. It is freely available as supplementary data online at MDPI.com. In order to use the QuantiIF macro, it is necessary to save the QuantIF.ijm file in the “Plugins” folder of ImageJ. The macro will then appear in the “Plugins” menu. When QuantIF is launched, the folder containing the images for analysis must be selected. Then, parameter values should be entered in a dialog box (Figure 1a), (i) the type/name of the specific staining, (ii) the staining threshold, and (iii) the size limits of nuclei. Once the parameters have been entered, the macro starts analyzing the images. They are first converted to 8-bit images, displaying 256 gray levels. Indeed, we recommend directly exporting images as 8-bit TIFF files, from the microscope software. The background of the images is then removed by running the efficient “Subtract Background” ImageJ command.

QuantIF relies on the “Analyze Particles” tool of ImageJ, which requires binary, black and white, images. For this reason, images are converted to binary masks by implementing the Huang’s fuzzy thresholding method [3]. An automatic threshold is set for DAPI staining images since strong and contrasted signals are expected for all these images (Figure 1b,c). In contrast, for immunostaining images, the automatic threshold is generally not applicable since some images may show no signal (in negative controls for instance). For this reason, a manual thresholding is implemented with the staining threshold value entered in the parameter’s dialog box (Figure 1a). The threshold value must range between 0 and 255; pixels with values under and above the threshold are converted to white and black, respectively (Figure 1d,e). The “Watershed” command is also applied to the DAPI staining mask in order to separate nearby nuclei [4]. However, it is important to avoid cell overconfluence to obtain interpretable results (see below). Furthermore, the commands “Dilate”, “Close”, and “Fill Holes” are applied to the mask of the specific staining in order to completely include the area corresponding to the nuclei. To analyze similar particles in the DAPI- and specific-staining masks and avoid counting autofluorescent debris, a new mask corresponding to the nuclei of immunostained cells is created. This is performed by executing the “Image Calculator” command and the “AND” operator using the DAPI- and specific-staining masks (Figure 1f). Finally, the total number of DAPI-stained and immunostained cells’ nuclei are counted by implementing the “Analyze Particles” tool to the DAPI staining mask (Figure 1g) and the immunostained cells’ nuclei mask (Figure 1h), respectively. The size limits for the nuclei entered in the parameters dialog box correspond to the minimum and maximum pixel area sizes that are taken into account to exclude anything that is not an object of interest. Additionally, to help exclude unwanted objects, roundness values have been set between 0.7 and 1.0.

After processing, the numbers of DAPI-stained nuclei and immunostained cells’ nuclei for each condition are saved as a “.xls” file in the folder that has been analyzed (Figure 1i). In addition, “Total Area”, “Average Size”, and “%Area” values are saved in the file. While “Total Area” values are not useful, the “Average Size” values can help in choosing the size limits for the nuclei that must be entered in the parameters dialog box. Furthermore, “%Area” values of the DAPI staining masks give an idea on cell confluence, which should not typically exceed 30% for optimal results. To help researchers find the best parameters for their analyses, the different masks can be saved in the folder that is being analyzed. To do so, the “//” symbols preceding the “saveAs” line commands must be deleted in the QuantIF.ijm file.

## 3. Discussion and Conclusions

QuantIF is a free, simple, and robust automated tool to estimate the proportion of virally infected cells after immunofluorescence. It is routinely used in our labs to quantify Hepatitis C Viral infections following detection of the E1 envelope glycoprotein that localizes predominantly to the endoplasmic reticulum in HCV-infected cells [5,6]. Similarly, we use it to evaluate Yellow Fever Virus infections using anti-E staining [5]. QuantIF is also used to quantify BKPyV and SV40 infections after detection of the VP1 or AgT proteins that show cytoplasmic and/or nuclear staining patterns (Figure 1d), as well as Coxsackievirus B4 infections using anti-VP1 staining [5]. QuantIF can also be used to quantify infection when using recombinant viruses expressing fluorescent reporter proteins [5]. Furthermore, it can serve many researchers for other applications that require counting labeled cells.

## Supplementary Materials

The following is available online at http://www.mdpi.com/1999-4915/11/2/165/s1, File S1: QuantIF.ijm.
